# Dissolvable Polyacrylamide Beads for High‐Throughput Droplet DNA Barcoding

**DOI:** 10.1002/advs.201903463

**Published:** 2020-02-20

**Authors:** Yongcheng Wang, Ting Cao, Jina Ko, Yinan Shen, Will Zong, Kuanwei Sheng, Wenjian Cao, Sijie Sun, Liheng Cai, Ying‐Lin Zhou, Xin‐Xiang Zhang, Chenghang Zong, Ralph Weissleder, David Weitz

**Affiliations:** ^1^ Wyss Institute for Biologically Inspired Engineering Harvard University Boston MA 02115 USA; ^2^ John A. Paulson School of Engineering and Applied Sciences and Department of Physics Harvard University Cambridge MA 02138 USA; ^3^ Department of Chemistry and Chemical Biology Harvard University Cambridge MA 02138 USA; ^4^ Beijing National Laboratory for Molecular Sciences (BNLMS) MOE Key Laboratory of Bioorganic Chemistry and Molecular Engineering College of Chemistry and Molecular Engineering Peking University Beijing 100871 China; ^5^ Center for Systems Biology Massachusetts General Hospital Harvard Medical School Boston MA 02114 USA; ^6^ Department of Molecular and Human Genetics Baylor College of Medicine Houston TX 77030 USA; ^7^ Department of Systems Biology Harvard Medical School Boston MA 02115 USA

**Keywords:** barcode beads, dissolvable hydrogels, droplet microfluidics, single‐cell sequencing

## Abstract

Droplet‐based single cell sequencing technologies, such as inDrop, Drop‐seq, and 10X Genomics, are catalyzing a revolution in the understanding of biology. Barcoding beads are key components for these technologies. What is limiting today are barcoding beads that are easy to fabricate, can efficiently deliver primers into drops, and thus achieve high detection efficiency. Here, this work reports an approach to fabricate dissolvable polyacrylamide beads, by crosslinking acrylamide with disulfide bridges that can be cleaved with dithiothreitol. The beads can be rapidly dissolved in drops and release DNA barcode primers. The dissolvable beads are easy to synthesize, and the primer cost for the beads is significantly lower than that for the previous barcoding beads. Furthermore, the dissolvable beads can be loaded into drops with >95% loading efficiency of a single bead per drop and the dissolution of beads does not influence reverse transcription or the polymerase chain reaction (PCR) in drops. Based on this approach, the dissolvable beads are used for single cell RNA and protein analysis.

## Graphical abstract

This work reports an approach to fabricate dissolvable polyacrylamide beads, by crosslinking acrylamide with disulfide bridges that can be cleaved with dithiothreitol. The beads can be rapidly dissolved in drops and release DNA barcode primers for droplet DNA barcoding, which demonstrate great potential for high‐throughput single cell sequencing.

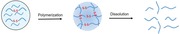

Droplet microfluidics have proven critical for next‐generation single‐cell analysis.[qv: 1] Coupled with high‐throughput sequencing, droplet‐based single cell sequencing technologies have been developed to analyze RNA,[qv: 2,3] DNA,[qv: 4,5] and even proteins[qv: 6,7] in single cells. To track single cells, barcodes need to be introduced, which is mainly achieved by adding synthetic DNA barcode primers to beads. For these applications, the beads are mostly made of biocompatible polymers, for example, polyacrylamide beads in inDrop,[qv: 2] hydroxylated methacrylic polymer beads used in Drop‐seq,[qv: 3] and polyacrylamide beads used in 10X Genomics.[qv: 8]

Each of these chemically different beads has their own advantages and shortcomings. The inDrop polyacrylamide bead can be closely packed in a microfluidic device channel to achieve more than 95% loading of single bead per drop.[qv: 2] However, in the inDrop system, UV light is necessary to release primers from the bead, which somewhat complicates bead fabrication and makes it less cost effective.[qv: 9] Another shortcoming is that UV light may introduce damage to DNA or RNA and skew the results.[qv: 10,11] The Drop‐seq system does not release primers from the bead and the reaction efficiency is low as reactions happen only near the surface of beads.[qv: 3] In the 10X Genomics platform, gel beads can be efficiently delivered into drops, but the comparably high cost and lack of flexibility in designing own primers on the beads are limitations for certain experiments.[qv: 8] What is thus missing today, are barcode beads that are easy to fabricate, can efficiently deliver primers into drops, and thus achieve high detection efficiency.

Here, we report a new approach to fabricate reversible polyacrylamide beads, which can be rapidly dissolved in drops and release DNA barcode primers in the presence of dithiothreitol (DTT; C_4_H_10_O_2_S_2_). As illustrated in **Figure** **1**A, a microfluidic droplet generation device is used to produce water in oil emulsion droplets of controlled size.[qv: 12,13] *N,N*′‐Bis(acryloyl)cystamine (BAC; C_10_H_16_N_2_O_2_S_2_), a reversible crosslinker,[qv: 14] reacts with acrylamide monomers (2‐propenamide; C_3_H_5_NO) to form a porous polyacrylamide hydrogel inside the water drops. The polyacrylamide hydrogel beads serve to i) carry primers by conjugating primers to the hydrogel, ii) deliver primers to individual drops, and iii) release primers by hydrogel dissolution. We used relatively low BAC concentration to make the hydrogel beads easy to dissolve while the beads could still maintain the morphology for carrying and delivering primers. The polyacrylamide bead can be rapidly dissolved in DTT by a thiol–disulfide exchange reaction.[qv: 14] DTT is a common redox reagent that is used to break down protein disulfide bonds and stabilize enzymes, and its addition to dissolve the beads does not influence reverse transcription or PCR. Acrydite‐modified DNA primers can be directly conjugated to the beads during polymerization. The conjugation yield of acrydite‐modified DNA primers to these polyacrylamide hydrogels is more than 50%.[qv: 2] They can be suspended into a homogeneous solution when beads are dissolved. The dissolution products of the beads are polymer fragments and (4S,5S)‐1,2‐dithiane‐4,5‐diol, as shown in Figure 1A. These products theoretically do not interfere with enzymatic reactions in drops. Using this technology, we show that beads can be completely dissolved in 3 min in 1 × 10^−3^ m DTT, and the primers on the beads can be rapidly suspended into solution. At the same time, we achieve >95% loading efficiency of a single bead per drop. We apply these barcoding beads to single cell RNA and protein analysis. As illustrated in Figure 1B, single cells and barcode beads are co‐encapsulated into drops. After barcoding of the molecular targets in drops, all DNA could be pooled together for sequencing, and single cell information could be retrieved by analyzing the cell barcodes.

**Figure 1:**
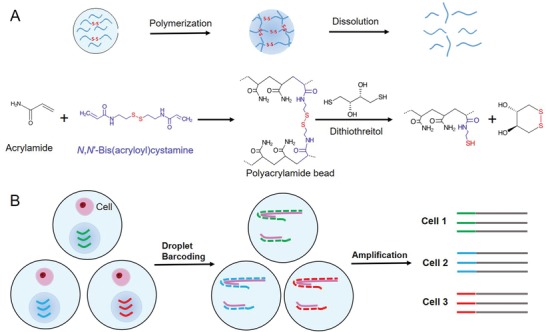
A) Schematic diagram of the dissolvable polyacrylamide beads synthesis and dissolution mechanism. Acrylamide monomers and *N*,*N*‐bis(acryloyl)cystamine crosslinker can react through C=C double bonds to form a polymer bead in the presence of ammonium persulfate (APS) and tetramethylethylenediamine (TEMED) at 70 °C overnight; the formed polyacrylamide bead can be dissolved when disulfide bonds are cleaved by DTT. B) Schematic diagram the dissolvable beads used for droplet‐based single cell analysis.

Homogeneous drops that contain acrylamide monomer and BAC are generated using a droplet generation device (Figure S1, Supporting Information). The size of the drop can be precisely controlled by the channel size of the device.[qv: 15] We choose conditions to create 70 µm drops to fabricate beads that have an ideal size for barcode addition and that can be encapsulated into drops. Polyacrylamide beads are formed by curing the droplets at 70 °C for overnight (Figure S2, Supporting Information). These beads can shrink or expand slightly in buffers with different salt concentrations. Increased ionic strength of the buffer usually causes the polyacrylamide beads to shrink. This is because at low ionic strength, the concentration of bound charges within the gel exceeds the concentration of salt in the external solution, and a large ion‐swelling pressure causes the gel to expand. As the ionic strength increases, the difference between the internal and external ion concentrations decreases and the gel shrinks.[qv: 16] Fourier Transform Raman Spectroscopy was performed to characterize the physicochemical property of the dissolvable polyacrylamide beads, as shown in Figure S3 (Supporting Information). The peaks at 484, 638, 1325, 1664, and 2926 cm^−1^ are correlated with (S—S), (C—S), (N—H), (C=O), and (CH_2_) bond stretching motions, respectively. The peaks at 1456 and 1614 cm^−1^ are correlated with (CH_2_) and (NH_2_) bond deformations, respectively.[qv: 17] These results indicate the successful synthesis of polyacrylamide gel with disulfide bonds.

We tested the dissolution time of the beads at different concentrations of DTT (**Figure** **2**A). The 70–90 µm beads can be completely dissolved in 13 min with 0.1 × 10^−3^ m DTT, in 3 min with 1 × 10^−3^ m DTT, and in 30 s with 10 × 10^−3^ m DTT. Figure 2B shows a set of microscopic images of the beads during the dissolving process with 1 × 10^−3^ m DTT (Video S1, Supporting Information). From 0 to 60 s, partial disulfide bonds are cleaved and the beads expand. From 60 to 120 s, more disulfide bonds are cleaved while breaking beads into fragments. At 180 s, beads can no longer be observed under the microscope.

**Figure 2:**
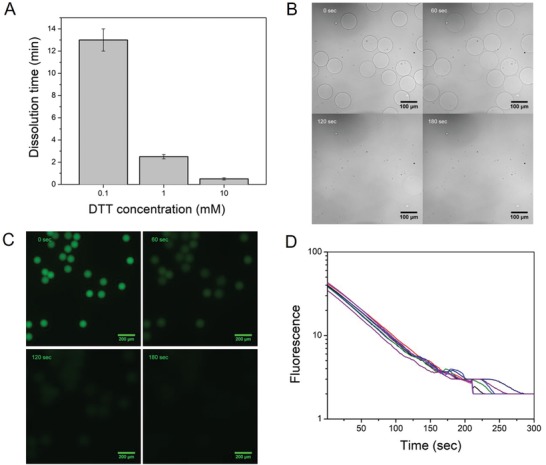
Fabrication and characterization of dissolvable polyacrylamide beads. A) Dissolution time of the beads with different DTT concentrations. B) Microscopic images of dissolving polyacrylamide beads at 0, 60, 120, and 180 s with 1 × 10^−3^ m DTT. C) Serial fluorescence images of FP‐labeled beads upon addition of 1 × 10^−3^ m DTT at 60, 120, and 180 s. The first image represents the original signal intensity before DTT addition. D) quantitative analysis of the fluorescence of eight representative beads versus time shows that the fluorescence of the bead is exponentially decaying. Each line represents a bead.

To study whether the dissolution of beads affects the viscosity of reaction solution, we measured the viscosity of solution with dissolved beads and without beads by using tracer particles. The viscosity of the solution with dissolved beads is (8.24 ± 0.72) × 10^4^ Pa s at 37 °C, which is only slightly higher (<3%) than that of the solution without beads, (8.03 ± 0.69) × 10^4^ Pa s. As a control, the solution with intact beads has similar viscosity to the solution without beads (Figure S4, Supporting Information). These measurements demonstrate that the dissolved beads have negligible influence on the physical property of the reaction solution.

To investigate its feasibility of primer release, we next conjugated acrydite‐modified DNA primers with 3′ polyT sequence (AP; Table S1, Supporting Information) into the hydrogel beads. A 5′ fluorescence‐labeled polyA primer (FP; Table S1, Supporting Information), complementary to polyT section of AP, was used to light the polyacrylamide hydrogel beads and track the primers on the beads. The primers on the beads are gradually dispersed into the solution along with the bead dissolution within 180 s (Figure 2C; Video S2, Supporting Information). Since there is an excess of DTT in the solution, the reaction rate is proportional to the amount of disulfide bonds (as shown in Equations (S1) and (S2) in the Supporting Information). Further quantitative analysis of the fluorescence intensity of eight representative beads during dissolution shows exponential dissolution and primer release (Figure 2D). This observation is consistent with our calculation of the reaction rate. Control experiments showed no fluorescence changes when FP‐labeled nondissolvable beads were used (Figure S5, Supporting Information), which indicates DTT has no direct effect on the intensity of FP. These results demonstrate that our designed dissolvable beads can rapidly release primers into a homogeneous solution and the release rate can be easily adjusted by changing DTT concentration in the reaction buffer.

Additionally, the acrydite primers used for the dissolvable beads only cost 3 $ nmol^−1^ for 1 µmol production scale from IDT (Integrated DNA Technologies, Inc.), while the acrydite photocleavable primers used for the inDrop beads cost 37 $ nmol^−1^ at the same production scale.[qv: 9] The 12 times lower barcode cost will likely translate into cheaper analysis or the ability to sequence more cells for the same price. More importantly, all bead operation processes can be performed under normal laboratory setting without the need of dark room operation, and the dissolution process is fully compatible with the molecular biology reactions in drops. These properties make dissolvable beads highly appealing for high‐throughput droplet DNA barcoding.

Droplet‐based single cell RNA sequencing has been widely used for analyzing cellular heterogeneity, understanding human biology and finding new drug targets.[qv: 18–20] To see if the dissolvable bead technology could similarly be used for single cell RNA sequencing, we performed the following proof‐of‐principle PCR experiment. Glyceraldehyde 3‐phosphate dehydrogenase (GAPDH) messenger RNA (mRNA), T‐cell receptor alpha locus (TCRA) mRNA, and total mRNA are measured from single human Jurkat T cells in drops by using the dissolvable beads that carry polyT primers for reverse transcription. PCR with GAPDH primer, TCRA primer, or total mRNA primer is performed to measure the complementary DNA (cDNA) product from the drop. The ability of loading one bead per drop is crucial for the recovery of single cell information. Almost all of the drops (>95%) contained single beads as we were able to closely pack the dissolvable beads in the channel of the microfluidic device to achieve the best possible loading (**Figure** **3**A).[qv: 21] Each bead carries ≈10^9^ primers, which is sufficient for capturing the transcriptome of an individual cell. After co‐encapsulation of single cells and the polyT primer conjugated dissolvable beads into drops, the polyT primers are released and bind with mRNA from the cell and start reverse transcription to make cDNA in drop. The cDNAs from the drops with cells could be amplified with targeted primers for GAPDH and TCRA, while the sample without cells did not show any amplification (Figure 3B), validating a successful reverse transcription using the polyT primers from the dissolvable beads. The size of the amplified PCR product was determined by agarose gel electrophoresis. The samples with cells show specific bands at 300 and 700 bp for GAPDH and TCRA, respectively (Figure 3C), which are consistent with our designed amplicon sizes. Moreover, SMART‐seq protocol with template switching oligo to amplify total mRNA is also performed with the dissolvable beads in drops.[qv: 22,23] The cDNA from the drops with cells could be amplified while the sample without cells did not show any amplification (Figure S6A, Supporting Information). Agarose gel electrophoresis shows that the sample with cells shows a smear that ranges from 200 to 1500 bp, which indicates amplicons from whole transcriptome in the cell (Figure S6B, Supporting Information). These results demonstrate the ability of the dissolvable beads to effectively analyze single cell RNA.

**Figure 3:**
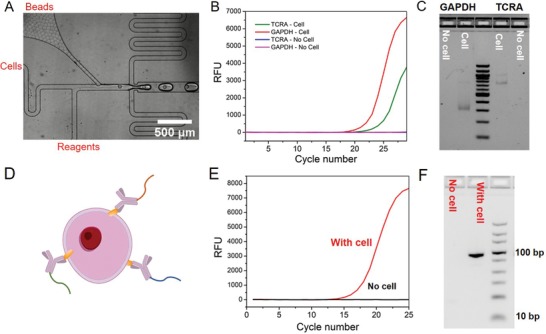
Single cell RNA and protein analysis with dissolvable beads. A) A microfluidic encapsulation device is used for cell and bead encapsulation. Almost all of the drops only contain one bead because of the close packing of the beads in the microfluidics channel. B) qPCR amplifications of TCRA and GAPDH mRNA from the samples with and without cell show the successful reverse transcription in drops by using dissolvable beads to deliver primers. C) Agarose gel electrophoresis image of the PCR‐amplified products shows specific bands for GAPDH and TCRA. D) Schematic of using the antibody–DNA conjugates to label the cell. E) qPCR data of the protein profiling for the samples with and without cells shows the successful amplification of antibody DNA on the cell. F) Agarose gel electrophoresis of the samples with and without cells confirms the amplification of antibody DNA.

Single cell protein analysis is of considerable scientific interest[qv: 24] and has been enabled by recent barcoding approaches.[qv: 25] New emerging methods, such as REAP‐seq,[qv: 7] have also been developed to quantify proteins from single cells by combining sequencing with droplet‐based microfluidic devices. Here we test the ability to use the dissolvable beads for single cell protein analysis. Based on earlier work,[qv: 25] we used antibody–DNA conjugates to investigate protein expression (Figure 3D). In this particular example, DNA serves two roles—as amplification and for future multiplexing. The protein expression of each cell can be converted to the DNA copy number.

As a proof of principle, we measured CD3 protein expression in human Jurkat T cells. Anti‐CD3 antibody was conjugated to DNA using trans‐cyclooctene (TCO)‐tetrazine (Tz) click chemistry, and the conjugation was validated using PAGE gel electrophoresis (Figure S7, Supporting Information). The antibody–DNA‐labeled cells were then encapsulated into drops containing the dissolvable bead. It is noteworthy that addition of DTT occurs after antibody hybridization, i.e., the DTT does not affect the DNA–antibody barcode prior to cellular binding. The amplicon is designed using the primer on the bead and the DNA conjugated to the antibody. Quantitative polymerase chain reaction (qPCR) amplification curves showed a successful amplification of the product in the presence of antibody–DNA‐labeled cells and no background without Jurkat cells (Figure 3E). Agarose gel electrophoresis image confirms that the size of the PCR‐amplified product is consistent with our designed full‐length amplicon (89 bp) (Figure 3F). Sanger sequencing of the PCR‐amplified product further validates that it has the same sequence with our design (Figure S8, Supporting Information). These results demonstrate the ability of the dissolvable beads to analyze single cell protein. Based on other data, it seems feasible to expand the technology to many other proteins, as has been done previously.[qv: 25]

In this work, we report a new method to fabricate polyacrylamide beads that can be rapidly dissolved in the presence of DTT for high‐throughput droplet‐based DNA barcoding. Imaging experiments show that 70–90 µm beads can be completely dissolved in 3 min with 1 × 10^−3^ m DTT, and the primers on the beads can be rapidly and evenly dispersed into the solution when the beads are dissolved. Coupling with droplet‐based microfluidic devices, we successfully demonstrate the ability of using the dissolvable beads as a high‐throughput barcoding cargo for single cell RNA and protein analysis.

The dissolvable beads deliver primers to each drop with high loading and reaction efficiency for both reverse transcription reaction and polymerase reaction in drops. The dissolvable bead technology overcomes multiple limitations of currently used barcode beads for single cell analysis by achieving both high loading and reaction efficiency in drops. Furthermore, the dissolvable beads can be easily synthesized at low cost. The beads can be dissolved in <3 min at high efficiency, a reaction that primarily depends on the DTT concentration. DTT is fully compatible with almost all molecular biology reactions in drops, which makes the technology easy to be integrated to existing molecular biology assays. These properties will make dissolvable polyacrylamide beads a very attractive tool for both high‐throughput single cell analysis and single molecular analysis.[qv: 26,27] This technology will open up opportunities to better understand and discover heterogeneous and rare molecular signatures that can be a key to diagnostics for various diseases.

## Conflict of Interest

The authors declare no conflict of interest.

## Supporting information

Supporting Information

Supplemental Video 1

Supplemental Video 2
