# Supplementary material for: Dissolvable Polyacrylamide Beads for High‐Throughput Droplet DNA Barcoding
Source: Adv Sci (Weinh). 2020 Feb 20;7(8):1903463. doi: 10.1002/advs.201903463 (PMC7175265; doi:10.1002/advs.201903463)
Supplement: Supplementary file 1 — Supporting Information [file ADVS-7-1903463-s001.pdf]

Copyright WILEY-VCH Verlag GmbH & Co. KGaA, 69469 Weinheim, Germany, 2020.

ADVANCED SCIENCE
Open Access

Supporting Information

for *Adv. Sci.,* DOI: 10.1002/advs.201903463

Dissolvable Polyacrylamide Beads for High-Throughput Droplet DNA Barcoding

Yongcheng Wang, Ting Cao, Jina Ko, Yinan Shen, Will Zong, Kuanwei Sheng, Wenjian Cao, Sijie Sun, Liheng Cai, Ying-Lin Zhou, Xin-Xiang Zhang, Chenghang Zong, Ralph Weissleder,* and David Weitz**

Copyright WILEY-VCH Verlag GmbH & Co. KGaA, 69469 Weinheim, Germany, 2020.

Supporting Information

Dissolvable polyacrylamide beads for high-throughput droplet DNA barcoding

Yongcheng Wang, Ting Cao, Jina Ko, Yinan Shen, Will Zong, Kuanwei Sheng, Wenjian Cao, Liheng Cai, Ying-Lin Zhou, Xin-Xiang Zhang, Chenghang Zong, Ralph Weissleder,* David Weitz**

Experimental Section

Buffer preparation: Tris-Buffered Saline–EDTA–Triton (TBSET) buffer, Tris–EDTA–Tween (TET) buffer, hybridization buffer and hydrogel bead wash buffer were prepared in advance and stored at room temperature. TBSET buffer contains 10 mM Tris–HCl (pH 8.0, Thermo Fisher Scientific, 15568025), 137 mM NaCl (Sigma-Aldrich, S3014-1KG), 270 mM KCl (Sigma-Aldrich, P9541-1KG), 10 mM EDTA (0.5 M; Thermo Fisher Scientific, 15575020) and 0.1% (vol/vol) Triton X-100 (Sigma-Aldrich, T8787-100ML). TET buffer contains 10 mM Tris–HCl (pH 8.0), 10 mM EDTA and 0.1% (vol/vol) Tween-20 (Fisher Scientific, BP337-100). Hybridization buffer contains 10 mM Tris–HCl (pH 8.0), 0.1 mM EDTA, 0.1% Tween-20 (vol/vol) and 330 mM KCl. Hydrogel bead wash buffer contains 10 mM Tris–HCl (pH 8.0), 0.1 mM EDTA and 0.1% Tween-20 (vol/vol). All buffers were filtered through 0.2-μm membranes (Complete Filtration Units; VWR, 10040-436) and could be stored for at least 6 months.

Microfluidic device fabrication: Our previous work has given a detailed microfluidic device fabrication procedure.[15] Computer Assisted Designs were firstly printed as photomasks to solidify a raised pattern as a master on a silicon wafer. Poly (dimethyl siloxane) (PDMS) base and curing agents (10:1, wt/wt) were mixed by Thinky Mixer and marked into channels using the master as a mold. Then acquired a PDMS slab and punched the inlet and outlet ports of this slab. Treated the channel side with oxygen plasma and bonded it with a glass slide to

obtain the microfluidic device. Dealt the channel surfaces with perfluorododecyltrichlorosilane for fluorophilic coating to produce monodisperse and reliable droplets before using this device.

Dissolvable beads fabrication: 500 µL solution mix was prepared containing 50 µL TBSET buffer, 30 µL 10% (w/v) APS (Sigma-Aldrich, A9164), 75 µL 40% (v/v) Acrylamide solution (Sigma-Aldrich, A4058-100ML), 20 µL 250 µM Acrydite-modified DNA primers (IDT, sequence in Table S1), 245 µL 0.8% (w/v) BAC (Sigma-Aldrich, A4929-5G) and 80 µL H_2O. Loaded this solution into a 1-mL syringe (Becton Dickinson, 309628). 1.5 mL carrier oil (RAN Biotechnologies, 008-FluoroSurfactant-2wtH-50G) and 6 µL of TEMED (Sigma-Aldrich, T9281-25ML) were mixed and loaded into a 3-mL syringe (Becton Dickinson, 309657). Connected these two syringes with inlets of the droplet generation device (Figure S1) by PE2 tubing (Scientific Commodities, BB31695-PE/2). Pumped the aqueous solution with 500 µL/h and the oil with 1000 µL/h. Collected the emulsion droplets at the outlet of the microfluidics chip. Covered the collected droplets with 200 µL mineral oil (Sigma-Aldrich, M5310-1L) and incubated at 70 °C for overnight. Centrifuged and discarded the carrier oil phase and mineral oil phase. 500 µL 20% (vol/vol) PFO (Alfa Aesar, B20156) in HFE 7500 (Novec 7500) was used to break the drops. The beads in the aqueous phase were washed with 1% Span-80 (Sigma-Aldrich, S6760-250ML) in hexane (Sigma-Aldrich, 227064-1L) twice and then with TBSET buffer 3 times. Filtered the beads through 70 µm cell strainer (Corning, 352350) and then stored them in TET buffer at 4 ^{o}C for up to 6 months Those beads have been washed at least 10 times before use to remove any remaining unreacted primers in the solution. FT-Raman Spectroscopy was recorded by NXR FT-Raman Module of Thermo Nicolet 6700 FT-IR Spectrophotometer (Thermo Fisher Scientific, USA). The gel bead was placed on a piece of aluminum foil and excited by the 1064 nm line of the laser source with a resolution of 8 cm^{-1}. Laser power was set to 2 W and scan number was 256.

Microscope imaging: For the bright field imaging, 1 µL of dissolvable beads and 9 µL of 1X ThermoPol buffer containing 1mM of DTT (100 mM, Thermo Fisher Scientific, 18064014) were mixed. Then the sample was loaded into a cell counting slide (NanoEnTek, EVS-050) immediately, and the beads dissolving process was recorded under fluorescent inverted microscope (Axio Observer Z1 Inverted Microscope, Carl Zeiss). For the fluorescent imaging of the primer release, 10 µL of dissolvable beads, 1 µL 100 mM FP (IDT, sequence in Table S1) and 89 µL Hybridization buffer were mixed and incubated for 10 minutes. Excess FP were removed by washing the beads 3 times by hybridization buffer. Then 1 µL of FP labelled dissolvable beads and 9 µL of 1X ThermoPol buffer containing 1mM of DTT were mixed and loaded onto a cell counting slide immediately for fluorescent imaging.

Viscosity measurement: To investigate the effect of dissolvable beads on the viscosity of solution, we mixed 1 µm PEG-coated tracer particles (F8823, ThermoFisher) with different solutions, and pipetted the solution into a 120 µm deep chamber, which was a double-side-sticky spacer with a hole of 9 mm in diameter (S24737, Life Technologies) sandwiched by two pieces of cover glass (2980-225, Corning). We observed the motion of these tracer particles using Zeiss Axio fluorescence microscope with a camera (Hamamatsu Flash4.0 V3) and an incubator at 37 ^{o}C, and took a video of 200 seconds at the frame rate of 100 frames per second. We extracted the trajectories of these particles and calculated their mean square displacement (MSD) and diffusion coefficient using Matlab. Given Stokes–Einstein equation, we thus obtained the viscosity of the solution. For each sample, we analyzed tens of particles and got an averaged viscosity of the sample.

Cell culture: Jurkat cells (ATCC® TIB-152™) were cultured in RPMI-1640 Medium (ATCC® 30-2001™), containing 10% FBS and 1X penicillin-streptomycin mix, in 5% CO_2 at 37 ^{o}C. After three days, harvested and split 1/5 cells out to be continuedly cultured in Non-Pyrogenic 6 well cell culture plates (Corning, USA). Centrifuged the remaining cell suspension with 300 g for 2 mins and discarded liquid supernatant. Washed cell pellet with

PBS for three times and then suspended in PBS by pipette. The cells were counted by Countess Automated Cell Counter (Invitrogen, USA). Human Embryonic Kidney 293 cells (HEK 293, ATCC® CRL-1573™) was cultured in Eagle's Minimum Essential Medium (EMEM, ATCC® 30-2003™), containing 10% FBS and 1x penicillin-streptomycin mix, in 5% CO_2 at 37 ^oC. HEK-293 cells are adhesive cells, so their cell treatment procedure is a little different from Jurkat cell. Discarded culture supernatant and washed the cells for three times with PBS. 200 µL trypsin was added for each well and incubated 3 min at 37 ^oC. Added 1 mL culture medium to stop trypsinization. The following steps were the same as Jurkat cell treatment.

Microfluidics encapsulation: Hydrogel beads were washed by hydrogel beads washing buffer for three times and then by 1X reaction buffer with 0.5% Triton for one time. Concentrated dissolvable beads were pulled into a PE2 tubing by a syringe until 3/4 tubing was occupied by the beads, which was then connected to a 1 mL syringe pre-filled with 200 µL HFE-7500 oil. Reagent phase was prepared based on different reactions and loaded to a 1 mL syringe that was also pre-filled with 200 µL HFE-7500 oil. 500 µL 10^5 cell/mL cells were prepared and suspended in PBS with 15% (vol/vol) OptiPrep (Sigma-Aldrich, D1556-250ML) and loaded into a 1 mL syringe as cell phase. 2 mL carrier oil was loaded into a 3 mL syringe. All syringes were connected to the microfluidics encapsulation device. The flow rates were 200 µL/h, 200 µL/h, 50 µL/h and 700 µL/h for the cell phase, reagent phase, beads phase and carrier oil phase, respectively. Tuned the flow rate of the beads phase to make sure most of the drops only contain one bead. The drops were collected when the bead encapsulation was stable.

Single cell RNA analysis: The reagents phase contains 32 µL 5X first strand buffer (Thermo Fisher Scientific, 18064014), 4 µL 10 mM dNTP (NEB, N0447L), 8 µL 10% (vol/vol) Triton X100, 12 µL 0.1 M DTT, 4 µL 40 µM template switching oligo (IDT, sequence in Table S1), 6 µL RNaseOUT (Thermo Fisher Scientific, 10777-019), 8 µL SuperScript II (Thermo Fisher

Scientific, 18064014) and 6 µL H_2O. 5-20 mM DTT usually exists in the reverse transcription reaction, which is sufficient to dissolve the beads. HEK 293 cell were suspended into PBS with 15% (vol/vol) OptiPrep. After encapsulation of the cells and beads, the drops were incubated at 42 °C for 90 min, 10 cycles of 50 °C for 2 min and 42 °C for 2 min, 15 min at 70 °C and hold at 4 °C on a Veriti 96 well Thermal Cycler (Applied Biosystem Inc). 20 µL 20% (vol/vol) PFO was added to 20 µL emulsion to break the drops. Transferred the top aqueous solution to a new tube, add 1 µL Thermolabile Exonuclease I (NEB, M0568S) to the solution and incubated at 37 °C for 4 min for primer digestion and 80 °C for 1 min for enzyme inactivation. Deep Vent® (exo-) DNA Polymerase (NEB, M0259S) was used to do PCR and qPCR amplification of the product. The qPCR mix contains 1 µL 10X ThermoPol buffer, 0.2 µL 10 mM dNTP, 0.5 µL 10 µM PCR primer (IDT, sequence in Table S1), 0.5 µL Evagreen, 0.1 µL DeepVent, 6.7 µL H_2O and 1 µL product. qPCR program (95 °C for 2 min; 25 cycles of 95 °C for 15 s, 60 °C for 15 and 72 °C for 3 min; final Extension at 72 °C for 5 min and hold at 4 °C) was run in the C1000 Thermal Cycler (Bio-Rad). The PCR mix had no Evagreen, and the other reagents were the same with qPCR mix. A 2% E-gel (Thermo Fisher Scientific, G501802) was used for analyzing the size of the PCR product and imaged by a FluorChem M system (ProteinSimple).

Antibody-DNA conjugation: BSA free anti-human CD3 antibody (BioXCell, BE0231) was buffer exchanged to biocarbonate buffer (pH8.4) using a 40k Zeba column (Thermo Fisher, 87765). The buffer-exchanged antibody was incubated with 10X excess TCO-PEG4-NHS Ester (Click Chesmitry Tools, A137-10) for 25mins at room temperature. After incubation, unlabeled TCO-PEG4-NHS Ester was removed using a 40k Zeba column. To check degree of labeling (DOL), the labelled antibody was incubated with 10X excess Cy3 Tetrazine (Click Chemistry Tools, 1018-1) for 25 mins at room temperature. Remaining Cy3 Tetrazine was removed using a 40k Zeba column. Cy3/Antibody ratio was measured using the Nanodrop UV/Vis mode (Thermo Fisher) at A550/A280. 1mM of amine-modified DNA oligo

(Integrated DNA Technologies) was buffer exchanged to borate buffer (pH8.5) using a 7k Zeba column (Thermo Fisher, 89878). The buffer-exchanged DNA oligo was incubated with 10X excess Methyltetrazine-PEG4-NHS Ester (Click Chemistry Tools, 1069-10) for 25 mins at room temperature. After incubation, unlabeled Tz-PEG4-NHS was removed using three 7k Zeba columns. To check DOL, the labelled DNA oligo was incubated with 4X excess AF488 TCO (Click Chemistry Tools, 1356-1) for 5 mins at room temperature. Remaining AF488 TCO was removed using a 7k Zeba column. AF488/DNA ratio was measured using the Nanodrop 1000 (Thermo Scientific) UV/Vis mode at A488/A260. Once TCO labelled antibody and Tz labelled DNA were prepared, they were mixed with appropriated DNA excess (Cy3/Antibody-0.5) and incubated for 45 mins at room temperature. Antibody amount was determined based on the need. Due to the usage of zeba columns (Thermo Fisher) for buffer exchange, we used 70μl of antibody (>1mg/ml) for a big batch and 12μl of antibody (>1mg/ml) for a small batch. Once antibody-DNA conjugates were made, they were stored in 4℃ for 1-2 weeks. The conjugation was validated using the NuPAGE 4-12% Bis-Tris Protein Gel (Thermo Fisher, NP0321BOX). Unconjugated antibody and DNA-conjugated antibody were incubated with 4X NuPAGE LDS Sample Buffer (Thermo Fisher, NP0007) without any reducing agents for 5 mins at 75 ℃ and loaded to the gel with Novex Sharp Pre-stained Protein Standard (Thermo Fisher, LC5800). The gel was run in 20X NuPAGE MOPS SDS Running Buffer (Thermo Fisher, NP0001) for one hour at 120 V.

Single cell protein analysis: The reagents phase contains 28 µL 10 mM dNTP, 9 µL 10% (vol/vol) Triton X100, 20 µL 0.1 M DTT, 20 µL 10X ThermoPol buffer (NEB, B9004S), 8 µL Bst 2.0 Warmstart (NEB, M0538S) and 16 µL H_2O. Jurkat cells were labelled with the antibody-DNA conjugate and suspended in PBS with 15% (vol/vol) OptiPrep. After encapsulation of cells and beads, the drop products were incubated at 60 °C for 2 hours and hold at 4 °C. 20 µL 20% (vol/vol) PFO was added to 20 µL emulsion to break the drops.

Transferred the top aqueous solution to a new tube. Added 1 µL Thermolabile Exonuclease I (NEB, M0568S) to the solution and incubated at 37 °C for 4 min for primer digestion and 80 °C for 1 min for enzyme inactivation. The qPCR mix contains 1 µL 10X ThermoPol buffer, 0.2 µL 10 mM dNTP, 0.5 µL 10 µM PCR primer 1 (IDT, sequence in Table S1), 0.5 µL 10 µM PCR primer 2 (IDT, sequence in Table S1), 0.5 µL Evagreen, 0.1 µL DeepVent, 6.2 µL H_2O and 1 µL product. qPCR program (95 °C for 2 min; 25 cycles of 95 °C for 15 s, 60 °C for 15 and 72 °C for 15 s; final Extension at 72 °C for 3 min and hold at 4 °C) was run in C1000 Thermal Cycler. The PCR mix had no Evagreen, and the other reagents were the same with qPCR mix. A 4% E-gel (Thermo Fisher Scientific, G401004) was used for analyzing the size of the PCR product. The PCR product was purified by 1.8X Ampure XP beads (Beckman Coulter, A63880) and sent to Genewiz for Sanger sequencing.

Table S1. Nucleic acid sequence used in this work

Oligo sequence used in RNA analysis	
AP (primer on beads)	/5Acryd/ATTATATATATGTGAGTGATGGTTGAGGATGTGTGGAGTTTTTTTTTTTTTTTTTTT
FP (Fluorescent probe)	56-FAM/ AAAAAAAAAAAAAAA
Template switching oligo	GTGAGTGATGGTTGAGGATGTGTGGAGTGrGrGrG
PCR primer	GTGAGTGATGGTTGAGGATGTGTGGAG
Oligo sequence used in protein analysis	
Antibody oligo	/5AmMC6/GAAGAGTTGTAGTGGAGGGTGTGTAGTACCGTT TCACCATACATCTTCACTCACATTCTC
Bead primer	/5Acryd/ATTATATATATGTGAGTGATGGTTGAGGATGTGTGGAGGAGAATGTGAGTGAAG ATGTATGGTGA

PCR primer 1	GTGAGTGATGGTTGAGGATGTGTGGAG
PCR primer 2	GAAGAGTTGTAGTGGAGGGTGTGTAGT
Full PCR amplicon	GTGAGTGATGGTTGAGGATGTGTGGAGGAGAATGTGAGTGAAGATGTATGGTGAAACG GTACTACACACCCTCCACTACAACTCTTC

Equation (1):

$$\frac{dN_{s-s}}{dt} = -kN_{s-s} \quad (1)$$

N_{s-s} is the amount of disulfide bonds in the beads, t is time, and k is a constant.

The solution to this equation is shown in **Equation (2):**

$$N_{s-s}(t) = N_0 e^{-kt} \quad (2)$$

$N_{s-s}(t)$ is the amount of disulfide bonds at time t and N_0 is the amount of disulfide bonds at time 0.

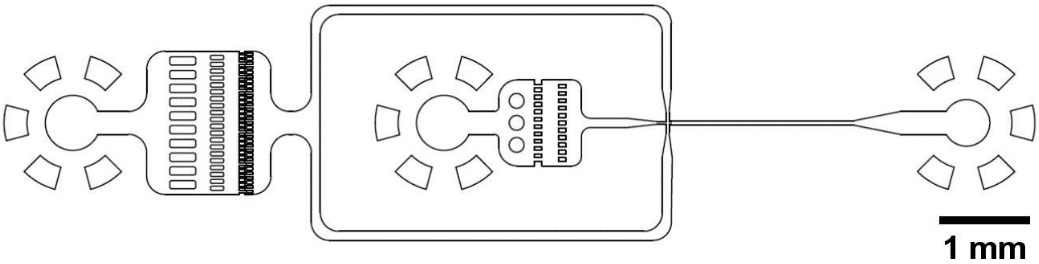

Figure S1. CAD design for the bead generation device.

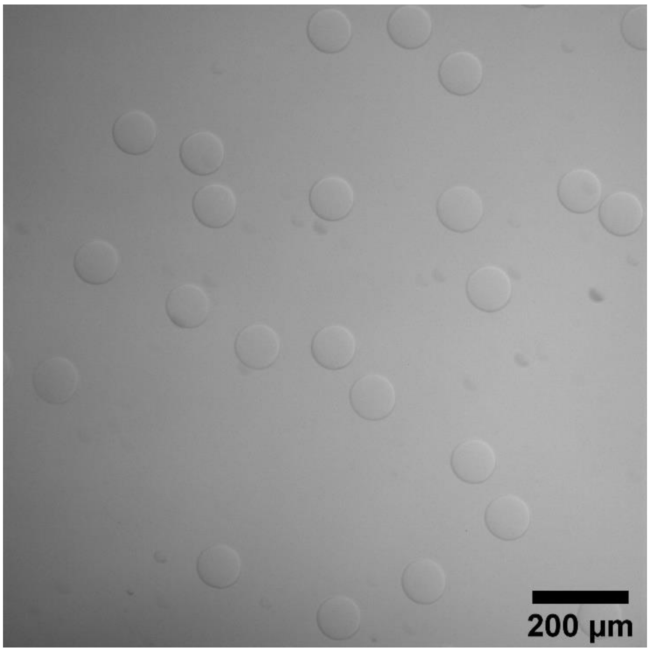

Figure S2. 70 µm dissolvable polyacrylamide beads.

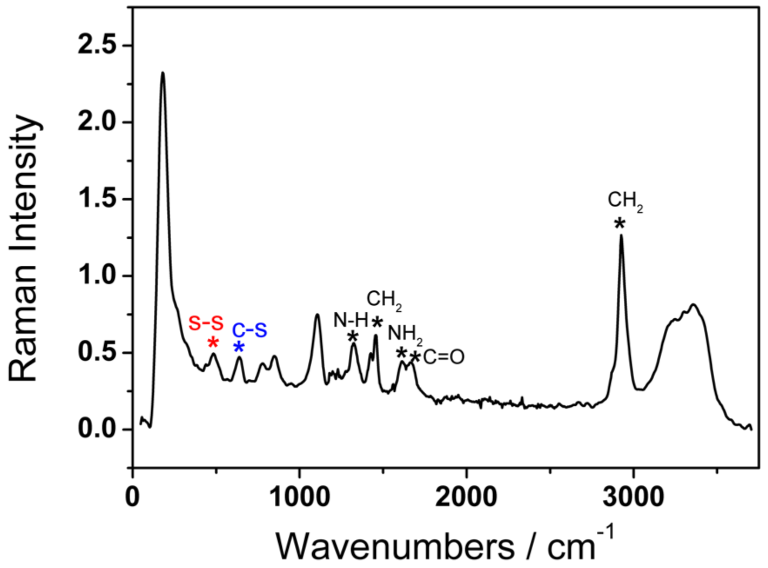

Figure S3. FT-Raman Spectroscopy of the dissolvable polyacrylamide bead. The peaks at 484 cm^{-1}, 638 cm^{-1}, 1325 cm^{-1}, 1664 cm^{-1} and 2926 cm^{-1} are correlated with (S-S), (C-S), (N-H), (C=O) and (CH_2) bond stretching motion, respectively. The peaks at 1456 cm^{-1} and 1614 cm^{-1} are correlated with (CH_2) and (NH_2) bond deformation, respectively.

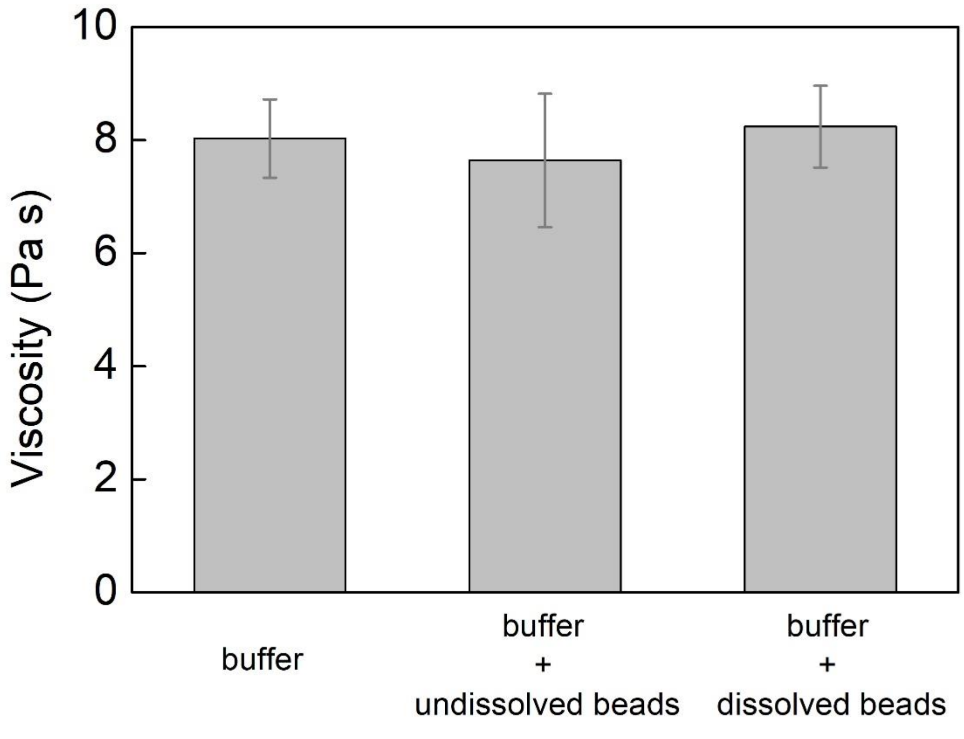

Figure S4. Viscosity of pure reaction buffer, undissolved beads in reaction buffer, and dissolved beads in reaction buffer.

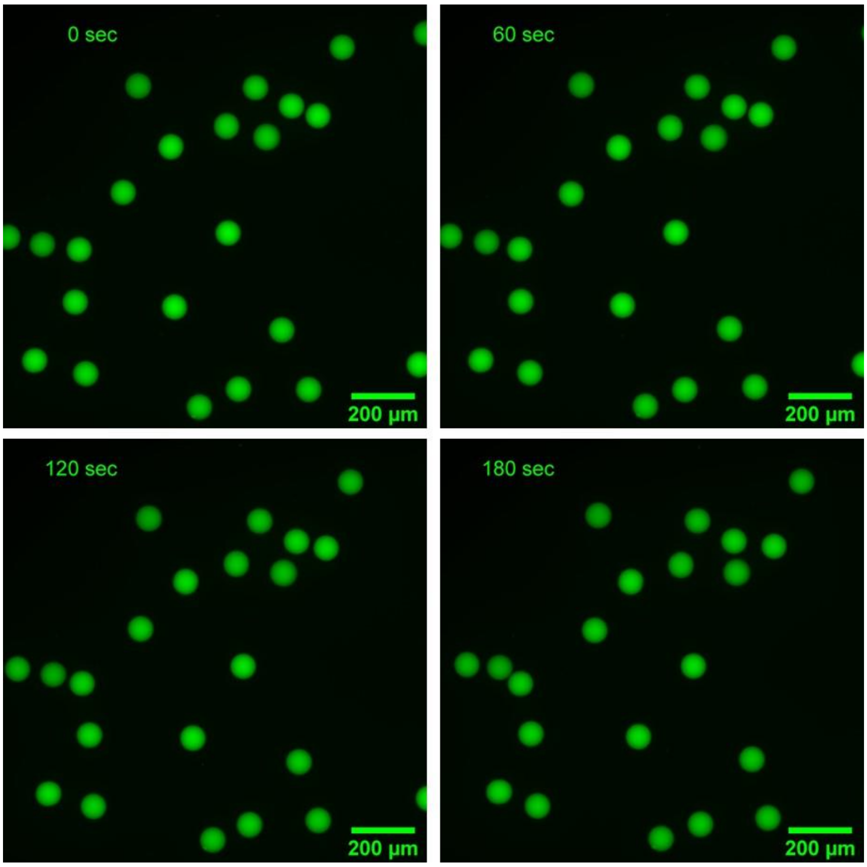

Figure S5. Serial fluorescence images of FP labeled non-dissolvable polyacrylamide beads upon addition of 1 mM DTT at 0 s 60 s, 120 s, and 180s.

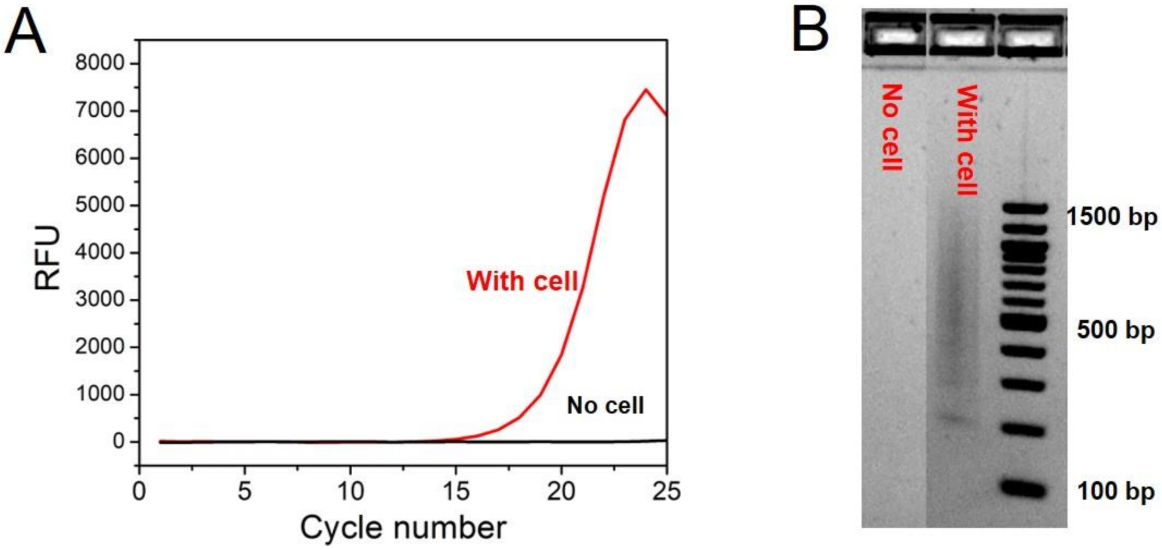

Figure S6. (A) qPCR amplification curves of the cDNA from the drops. The red curve is the sample with cell, while the black curve is the negative control sample without cell. (B) Agarose gel electrophoresis image of the PCR amplified transcriptome products. The sample with cell shows a smear indicates the amplicons are from the transcriptome in the cell.

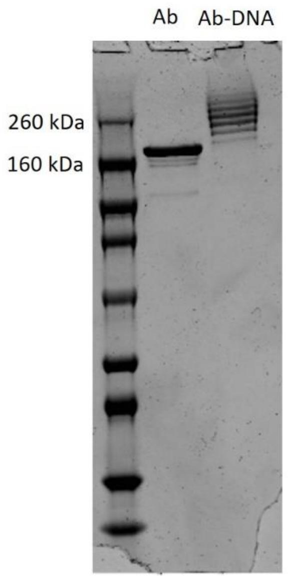

Figure S7. Antibody-DNA conjugates for protein profiling. DNA conjugated antibody shows multiple shifted bands compared to unlabeled antibody. The multiple shifted bands indicate different degree of labeling of DNA attached to antibody.

```
         1                                                50
Template --GTGAGTGATGGTTGAGGATGTGTGGAGGAGAATGTGAGTGAAGATGTA
primer 1 NNNNNAGTGATGGTTGAGGATGTGTGGAGGAGAATGTGAGNNAAGNTGTN
primer 2 -----------------------------NNNNNNNNNNANTGANNNNTA

..............................................................

         51                                      91
Template TGGTGAAACGGTACTACACACCCTCCACTACAACTCTTC--
primer 1 NNNNNNNNNNNN-----------------------------
primer 2 T-GNTGAACGGTACTACACACCCTCCACTACAACTCTTNAN

..............................................................
```

Figure S8. Sanger sequencing result for the droplet barcoded antibody DNA amplicon.
